# (*E*)-*N*′-(4-Hydroxy­benzyl­idene)-4-hydroxy­benzohydrazide methanol solvate

**DOI:** 10.1107/S1600536809020078

**Published:** 2009-06-06

**Authors:** Cong-Ming Li, Hong-Yan Ban

**Affiliations:** a College of Science, Shenyang University, Shenyang 110044, People’s Republic of China; b School of Chemical Engineering, University of Science and Technology Liaoning, Anshan 114051, People’s Republic of China

## Abstract

The title compound, C_14_H_12_N_2_O_3_·CH_4_O, consists of a Schiff base mol­ecule and a methanol mol­ecule of crystallization. The Schiff base mol­ecule is nearly planar, the dihedral angle between the planes of the two benzene rings being 7.2 (2)°. The mol­ecule exists in the *trans* configuration with respect to the methyl­idene unit. In the crystal structure, the Schiff base and methanol mol­ecules are linked through O—H⋯O, N—H⋯O and O—H⋯N hydrogen bonds, forming a three-dimensional network.

## Related literature

For the biological activity of hydrazones, see: Zhong *et al.* (2007 ▶); Raj *et al.* (2007 ▶); Jimenez-Pulido *et al.* (2008 ▶). For related crystal structures, see: Ban & Li (2008*a* ▶,*b* ▶); Li & Ban (2009*a* ▶,*b* ▶); Yehye *et al.* (2008 ▶); Fun, Patil, Jebas *et al.* (2008 ▶); Fun, Patil, Rao *et al.* (2008 ▶); Yang *et al.* (2008 ▶); Ejsmont *et al.* (2008 ▶).
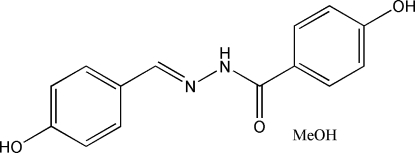


## Experimental

### 

#### Crystal data

C_14_H_12_N_2_O_3_·CH_4_O
*M* *_r_* = 288.30
Monoclinic, 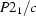

*a* = 12.927 (1) Å
*b* = 9.277 (1) Å
*c* = 11.946 (2) Å
β = 100.147 (1)°
*V* = 1410.2 (3) Å^3^
*Z* = 4
Mo *K*α radiation
μ = 0.10 mm^−1^
*T* = 298 K
0.30 × 0.30 × 0.28 mm

#### Data collection

Bruker SMART CCD area-detector diffractometer
Absorption correction: multi-scan (*SADABS*; Sheldrick, 1996 ▶) *T*_min_ = 0.971, *T*_max_ = 0.973
8435 measured reflections
3064 independent reflections
2382 reflections with *I* > 2σ(*I*)
*R*_int_ = 0.023

#### Refinement

*R*[*F* ^2^ > 2σ(*F* ^2^)] = 0.040
*wR*(*F* ^2^) = 0.118
*S* = 1.06
3064 reflections
197 parameters
1 restraint
H atoms treated by a mixture of independent and constrained refinement
Δρ_max_ = 0.22 e Å^−3^
Δρ_min_ = −0.17 e Å^−3^

### 

Data collection: *SMART* (Bruker, 1998 ▶); cell refinement: *SAINT* (Bruker, 1998 ▶); data reduction: *SAINT*; program(s) used to solve structure: *SHELXS97* (Sheldrick, 2008 ▶); program(s) used to refine structure: *SHELXL97* (Sheldrick, 2008 ▶); molecular graphics: *SHELXTL* (Sheldrick, 2008 ▶); software used to prepare material for publication: *SHELXTL*.

## Supplementary Material

Crystal structure: contains datablocks global, I. DOI: 10.1107/S1600536809020078/wn2329sup1.cif

Structure factors: contains datablocks I. DOI: 10.1107/S1600536809020078/wn2329Isup2.hkl

Additional supplementary materials:  crystallographic information; 3D view; checkCIF report

## Figures and Tables

**Table 1:** Hydrogen-bond geometry (Å, °)

*D*—H⋯*A*	*D*—H	H⋯*A*	*D*⋯*A*	*D*—H⋯*A*
O1—H1⋯O2^i^	0.82	1.90	2.6877 (14)	160
O1—H1⋯N1^i^	0.82	2.61	3.1521 (16)	125
O3—H3⋯O1^ii^	0.82	1.90	2.7156 (15)	172
O4—H4⋯O2	0.82	1.95	2.7629 (15)	173
N2—H2*A*⋯O4^iii^	0.892 (9)	2.100 (11)	2.9695 (16)	164.6 (19)

Symmetry codes: (i) 
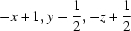
; (ii) 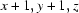; (iii) 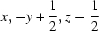.

**Fig. 1.**
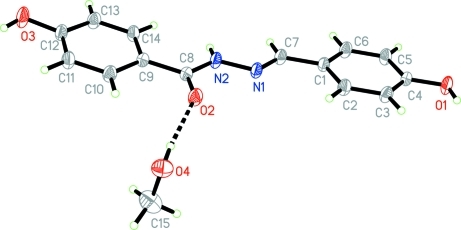
The molecular structure of the title compound, showing 30% probability displacement ellipsoids for the non-hydrogen atoms. Hydrogen atoms are shown as spheres of arbitrary radius. The dashed line indicates a hydrogen bond.

**Fig. 2.**
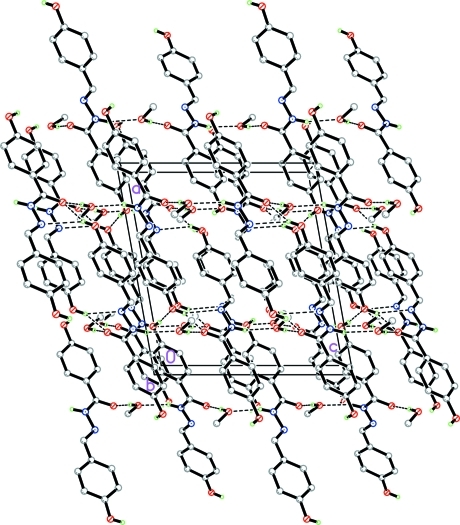
The packing diagram, viewed along the b axis. Hydrogen bonds are shown as dashed lines. Hydrogen atoms not involved in hydrogen bonding have been omitted.

## supplementary crystallographic information

### Comment

Schiff bases derived from the condensation of aldehydes with hydrazides have been shown to possess excellent biological activities (Zhong *et al.*, 2007; Raj *et al.*, 2007; Jimenez-Pulido *et al.*, 2008). Due to the easy synthesis of such compounds, many Schiff bases have been synthesized and structurally characterized (Yehye *et al.*, 2008; Fun, Patil, Jebas *et al.* (2008); Fun, Patil, Rao *et al.* (2008); Yang *et al.*, 2008; Ejsmont *et al.*, 2008). Recently, we have reported a few such compounds (Ban & Li, 2008a,b; Li & Ban, 2009a,b). In this paper, we report the crystal structure of the new title compound.

In the structure of the title compound (Fig. 1) the Schiff base molecule is nearly planar, the dihedral angle between the two benzene rings being 7.2 (2)°. The molecule exists in a *trans* configuration with respect to the methylidene unit. The torsion angle C7—N1—N2—C8 is 0.3 (2)°.

In the crystal structure, the Schiff base molecules and the methanol molecules are linked through intermolecular O—H···O, N—H···O and O—H···N hydrogen bonds (Table 1), forming a three dimensional network (Fig. 2).

### Experimental

The compound was prepared by refluxing 4-hydroxybenzaldehyde (1.0 mol) with 4-hydroxybenzohydrazide (1.0 mol) in methanol (100 ml). Excess methanol was removed from the mixture by distillation. The colorless solid product was filtered and washed three times with methanol. Colorless block crystals of the title compound were obtained from a methanol solution by slow evaporation in air.

### Refinement

H2A was located in a difference Fourier map and refined isotropically, with the N—H distance restrained to 0.90 (1)Å. Other H atoms were placed in calculated positions (C—H = 0.93 - 0.96 Å, O—H = 0.82 Å) and refined as riding with U_iso_(H) = 1.2U_eq_(C) and 1.5U_eq_(O and methyl). A rotating group model was used for the methyl group of the methanol.

### Crystal data

**Table:**

C_14_H_12_N_2_O_3_·CH_4_O	*F*(000) = 608
*M**_r_* = 288.30	*D*_x_ = 1.358 Mg m^−^^3^
Monoclinic, *P*2_1_/*c*	Mo *K*α radiation, λ = 0.71073 Å
Hall symbol: -P 2ybc	Cell parameters from 2955 reflections
*a* = 12.927 (1) Å	θ = 2.7–29.4°
*b* = 9.277 (1) Å	µ = 0.10 mm^−^^1^
*c* = 11.946 (2) Å	*T* = 298 K
β = 100.147 (1)°	Block, colorless
*V* = 1410.2 (3) Å^3^	0.30 × 0.30 × 0.28 mm
*Z* = 4	

### Data collection

**Table:**

Bruker SMART CCD area-detector diffractometer	3064 independent reflections
Radiation source: fine-focus sealed tube	2382 reflections with *I* > 2σ(*I*)
graphite	*R*_int_ = 0.023
ω scans	θ_max_ = 27.0°, θ_min_ = 1.6°
Absorption correction: multi-scan (*SADABS*; Sheldrick, 1996)	*h* = −10→16
*T*_min_ = 0.971, *T*_max_ = 0.973	*k* = −11→11
8435 measured reflections	*l* = −15→14

### Refinement

**Table:**

Refinement on *F*^2^	Secondary atom site location: difference Fourier map
Least-squares matrix: full	Hydrogen site location: inferred from neighbouring sites
*R*[*F*^2^ > 2σ(*F*^2^)] = 0.040	H atoms treated by a mixture of independent and constrained refinement
*wR*(*F*^2^) = 0.118	*w* = 1/[σ^2^(*F*_o_^2^) + (0.0556*P*)^2^ + 0.2974*P*] where *P* = (*F*_o_^2^ + 2*F*_c_^2^)/3
*S* = 1.06	(Δ/σ)_max_ < 0.001
3064 reflections	Δρ_max_ = 0.22 e Å^−^^3^
197 parameters	Δρ_min_ = −0.17 e Å^−^^3^
1 restraint	Extinction correction: *SHELXL97* (Sheldrick, 2008), Fc^*^=kFc[1+0.001xFc^2^λ^3^/sin(2θ)]^-1/4^
Primary atom site location: structure-invariant direct methods	Extinction coefficient: 0.0112 (19)

### Special details

**Table:**

Geometry. All esds (except the esd in the dihedral angle between two l.s. planes) are estimated using the full covariance matrix. The cell esds are taken into account individually in the estimation of esds in distances, angles and torsion angles; correlations between esds in cell parameters are only used when they are defined by crystal symmetry. An approximate (isotropic) treatment of cell esds is used for estimating esds involving l.s. planes.
Refinement. Refinement of F^2^ against ALL reflections. The weighted R-factor wR and goodness of fit S are based on F^2^, conventional R-factors R are based on F, with F set to zero for negative F^2^. The threshold expression of F^2^ > 2sigma(F^2^) is used only for calculating R-factors(gt) etc. and is not relevant to the choice of reflections for refinement. R-factors based on F^2^ are statistically about twice as large as those based on F, and R- factors based on ALL data will be even larger.

### Fractional atomic coordinates and isotropic or equivalent isotropic displacement parameters (Å^2^)

**Table:**

	*x*	*y*	*z*	*U*_iso_*/*U*_eq_	
N1	0.70137 (9)	0.02841 (13)	0.10898 (10)	0.0378 (3)	
N2	0.78657 (9)	0.08908 (13)	0.07052 (10)	0.0383 (3)	
O1	0.30453 (8)	−0.37263 (12)	0.13979 (9)	0.0485 (3)	
H1	0.2840	−0.3402	0.1959	0.073*	
O2	0.80648 (8)	0.24758 (12)	0.21426 (9)	0.0475 (3)	
O3	1.21247 (9)	0.40526 (14)	0.01129 (11)	0.0588 (4)	
H3	1.2348	0.4747	0.0508	0.088*	
O4	0.79337 (10)	0.50038 (13)	0.33324 (9)	0.0565 (3)	
H4	0.8007	0.4286	0.2953	0.085*	
C1	0.56666 (10)	−0.15101 (15)	0.07692 (11)	0.0352 (3)	
C2	0.52247 (11)	−0.11701 (16)	0.17196 (12)	0.0413 (4)	
H2	0.5519	−0.0441	0.2208	0.050*	
C3	0.43564 (11)	−0.19027 (17)	0.19437 (12)	0.0410 (3)	
H3A	0.4071	−0.1669	0.2582	0.049*	
C4	0.39100 (10)	−0.29833 (15)	0.12210 (11)	0.0342 (3)	
C5	0.43415 (12)	−0.33417 (17)	0.02789 (13)	0.0429 (4)	
H5	0.4046	−0.4072	−0.0208	0.052*	
C6	0.52155 (11)	−0.26075 (17)	0.00652 (12)	0.0432 (4)	
H6	0.5507	−0.2857	−0.0566	0.052*	
C7	0.65706 (11)	−0.07482 (16)	0.04877 (12)	0.0392 (3)	
H7	0.6834	−0.1026	−0.0156	0.047*	
C8	0.83705 (10)	0.19889 (15)	0.12912 (12)	0.0347 (3)	
C9	0.93302 (10)	0.25557 (14)	0.09212 (11)	0.0341 (3)	
C10	0.98624 (11)	0.36828 (16)	0.15352 (13)	0.0420 (4)	
H10	0.9590	0.4089	0.2133	0.050*	
C11	1.07881 (12)	0.42096 (17)	0.12748 (13)	0.0443 (4)	
H11	1.1135	0.4966	0.1694	0.053*	
C12	1.11995 (11)	0.36095 (16)	0.03881 (13)	0.0403 (3)	
C13	1.06694 (12)	0.25061 (17)	−0.02465 (13)	0.0451 (4)	
H13	1.0938	0.2116	−0.0853	0.054*	
C14	0.97452 (11)	0.19819 (16)	0.00163 (12)	0.0413 (4)	
H14	0.9394	0.1238	−0.0414	0.050*	
C15	0.74278 (19)	0.6095 (2)	0.26152 (16)	0.0737 (6)	
H15A	0.7498	0.6999	0.3012	0.111*	
H15B	0.6696	0.5862	0.2397	0.111*	
H15C	0.7745	0.6166	0.1948	0.111*	
H2A	0.8014 (16)	0.059 (2)	0.0042 (11)	0.080*	

### Atomic displacement parameters (Å^2^)

**Table:**

	*U*^11^	*U*^22^	*U*^33^	*U*^12^	*U*^13^	*U*^23^
N1	0.0316 (6)	0.0421 (7)	0.0427 (6)	−0.0080 (5)	0.0150 (5)	0.0011 (5)
N2	0.0346 (6)	0.0438 (7)	0.0406 (7)	−0.0115 (5)	0.0174 (5)	−0.0034 (5)
O1	0.0437 (6)	0.0549 (7)	0.0533 (7)	−0.0208 (5)	0.0262 (5)	−0.0121 (5)
O2	0.0476 (6)	0.0488 (6)	0.0528 (6)	−0.0110 (5)	0.0271 (5)	−0.0127 (5)
O3	0.0459 (6)	0.0687 (8)	0.0696 (8)	−0.0257 (6)	0.0318 (6)	−0.0206 (6)
O4	0.0673 (8)	0.0592 (7)	0.0440 (6)	0.0072 (6)	0.0124 (5)	−0.0073 (5)
C1	0.0315 (7)	0.0377 (7)	0.0382 (7)	−0.0047 (6)	0.0111 (6)	0.0015 (6)
C2	0.0394 (8)	0.0447 (8)	0.0421 (8)	−0.0109 (6)	0.0135 (6)	−0.0096 (6)
C3	0.0390 (8)	0.0501 (9)	0.0379 (7)	−0.0068 (6)	0.0178 (6)	−0.0062 (6)
C4	0.0297 (7)	0.0365 (7)	0.0387 (7)	−0.0042 (5)	0.0123 (6)	0.0025 (6)
C5	0.0427 (8)	0.0450 (8)	0.0445 (8)	−0.0149 (7)	0.0170 (6)	−0.0126 (7)
C6	0.0441 (8)	0.0498 (9)	0.0405 (8)	−0.0097 (7)	0.0213 (7)	−0.0085 (7)
C7	0.0364 (7)	0.0452 (8)	0.0392 (7)	−0.0076 (6)	0.0154 (6)	−0.0015 (6)
C8	0.0319 (7)	0.0348 (7)	0.0395 (7)	−0.0012 (6)	0.0122 (6)	0.0009 (6)
C9	0.0306 (7)	0.0351 (7)	0.0382 (7)	−0.0029 (5)	0.0106 (6)	0.0015 (6)
C10	0.0396 (8)	0.0460 (8)	0.0440 (8)	−0.0073 (6)	0.0174 (6)	−0.0085 (6)
C11	0.0404 (8)	0.0463 (8)	0.0487 (9)	−0.0146 (7)	0.0144 (7)	−0.0118 (7)
C12	0.0339 (7)	0.0438 (8)	0.0462 (8)	−0.0084 (6)	0.0148 (6)	0.0001 (6)
C13	0.0423 (8)	0.0507 (9)	0.0475 (8)	−0.0093 (7)	0.0217 (7)	−0.0115 (7)
C14	0.0390 (8)	0.0413 (8)	0.0462 (8)	−0.0117 (6)	0.0146 (6)	−0.0097 (6)
C15	0.1079 (17)	0.0625 (12)	0.0508 (11)	−0.0016 (11)	0.0144 (11)	0.0044 (9)

### Geometric parameters (Å, °)

**Table:**

N1—C7	1.2718 (18)	C5—C6	1.3810 (19)
N1—N2	1.3853 (15)	C5—H5	0.9300
N2—C8	1.3390 (18)	C6—H6	0.9300
N2—H2A	0.892 (9)	C7—H7	0.9300
O1—C4	1.3611 (16)	C8—C9	1.4846 (18)
O1—H1	0.8200	C9—C10	1.3881 (19)
O2—C8	1.2395 (16)	C9—C14	1.3943 (19)
O3—C12	1.3587 (16)	C10—C11	1.3780 (19)
O3—H3	0.8200	C10—H10	0.9300
O4—C15	1.410 (2)	C11—C12	1.383 (2)
O4—H4	0.8200	C11—H11	0.9300
C1—C6	1.382 (2)	C12—C13	1.382 (2)
C1—C2	1.3939 (19)	C13—C14	1.376 (2)
C1—C7	1.4549 (18)	C13—H13	0.9300
C2—C3	1.3782 (19)	C14—H14	0.9300
C2—H2	0.9300	C15—H15A	0.9600
C3—C4	1.381 (2)	C15—H15B	0.9600
C3—H3A	0.9300	C15—H15C	0.9600
C4—C5	1.3817 (19)		
			
C7—N1—N2	115.06 (11)	O2—C8—N2	120.65 (12)
C8—N2—N1	118.58 (11)	O2—C8—C9	121.28 (13)
C8—N2—H2A	122.8 (14)	N2—C8—C9	118.01 (11)
N1—N2—H2A	118.4 (14)	C10—C9—C14	118.34 (12)
C4—O1—H1	109.5	C10—C9—C8	118.16 (12)
C12—O3—H3	109.5	C14—C9—C8	123.46 (12)
C15—O4—H4	109.5	C11—C10—C9	121.15 (13)
C6—C1—C2	118.21 (12)	C11—C10—H10	119.4
C6—C1—C7	119.15 (12)	C9—C10—H10	119.4
C2—C1—C7	122.64 (13)	C10—C11—C12	119.76 (14)
C3—C2—C1	120.74 (13)	C10—C11—H11	120.1
C3—C2—H2	119.6	C12—C11—H11	120.1
C1—C2—H2	119.6	O3—C12—C13	117.65 (13)
C2—C3—C4	120.09 (12)	O3—C12—C11	122.48 (13)
C2—C3—H3A	120.0	C13—C12—C11	119.87 (13)
C4—C3—H3A	120.0	C14—C13—C12	120.20 (13)
O1—C4—C3	122.32 (12)	C14—C13—H13	119.9
O1—C4—C5	117.71 (13)	C12—C13—H13	119.9
C3—C4—C5	119.97 (12)	C13—C14—C9	120.66 (13)
C6—C5—C4	119.52 (13)	C13—C14—H14	119.7
C6—C5—H5	120.2	C9—C14—H14	119.7
C4—C5—H5	120.2	O4—C15—H15A	109.5
C5—C6—C1	121.46 (13)	O4—C15—H15B	109.5
C5—C6—H6	119.3	H15A—C15—H15B	109.5
C1—C6—H6	119.3	O4—C15—H15C	109.5
N1—C7—C1	122.36 (13)	H15A—C15—H15C	109.5
N1—C7—H7	118.8	H15B—C15—H15C	109.5
C1—C7—H7	118.8		

### Hydrogen-bond geometry (Å, °)

**Table:**

*D*—H···*A*	*D*—H	H···*A*	*D*···*A*	*D*—H···*A*
O1—H1···O2^i^	0.82	1.90	2.6877 (14)	160
O1—H1···N1^i^	0.82	2.61	3.1521 (16)	125
O3—H3···O1^ii^	0.82	1.90	2.7156 (15)	172
O4—H4···O2	0.82	1.95	2.7629 (15)	173
N2—H2A···O4^iii^	0.89 (1)	2.10 (1)	2.9695 (16)	165 (2)

Symmetry codes: (i) −*x*+1, *y*−1/2, −*z*+1/2; (ii) *x*+1, *y*+1, *z*; (iii) *x*, −*y*+1/2, *z*−1/2.
